# Essential oils compromise tegumental integrity in *Sparicotyle chrysophrii*: Insights from SEM and histological analyses

**DOI:** 10.1007/s00436-026-08708-z

**Published:** 2026-06-05

**Authors:** Teresa Pirollo, Perla Tedesco, Andrea Gustinelli, Ana Leon, Oswaldo Palenzuela, Itziar Estensoro, Monica Caffara

**Affiliations:** 1 Department of Veterinary Medical Sciences, Alma Mater Studiorum University of Bologna, Via Tolara di Sopra 50, 40064 Ozzano Emilia, Bologna Italy; 2 Instituto de Acuicultura Torre de la Sal, Consejo Superior de Investigaciones Científicas(IATS, CSIC), Castellón, Spain; 3 Doctorate program in Science and Technology in Animal Production, Universitat Politècnica de València, València, Spain

**Keywords:** *Sparicotyle chrysophrii*, essential oils, Scanning electron microscopy, Histology

## Abstract

This study investigates the morphological effects of various essential oils (EOs) on the monogenean *Sparicotyle chrysophrii*, a major gill parasite of gilthead sea bream (*Sparus aurata*), through scanning electron microscopy (SEM) and histological analyses. Adult parasites obtained from experimental infections were exposed *in vitro* to different EOs at concentrations ranging from 62.5 to 500 ppm. *Cinnamomum zeylanicum*, *Origanum vulgare*, *Thymus vulgaris*, and a commercial blend (Mix Gill) exhibited the highest antiparasitic efficacy, achieving complete mortality at 500 ppm within one hour. SEM observations revealed extensive tegumental disruption, including surface erosion, deep wrinkling, and clamp detachment, which increased in severity with EO concentration and exposure time. Histological analyses confirmed widespread tissue degeneration, characterized by vacuolization, cytoplasmic rarefaction, and loss of the neodermal and muscular organization. Even at sublethal concentrations, lesions consistent with swelling, vesicle formation, and tegumental disorganization indicated progressive degeneration. These structural alterations suggest that EOs compromise the parasite’s external integrity, leading to functional collapse of the tegument and haptor. The results support a time- and concentration-dependent mechanism, likely involving disruption of membrane integrity and osmotic imbalance. Collectively, to the best of our knowledge, this is among the first studies to provide detailed ultrastructural evidence of EO-induced damage in *S. chrysophrii* and highlights their potential as eco-friendly antiparasitic agents for aquaculture. Further studies integrating ultrastructural and biochemical analyses are warranted to elucidate the molecular pathways underlying these effects.

## Introduction

*Sparicotyle chrysophrii* is a polyophistocotylean haematophagus parasite that specifically infect gills of gilthead sea bream (*Sparus aurata* L.), both in wild and aquaculture settings throughout the Mediterranean Sea. This parasite is recognized as the causative agent of sparicotylosis, an enzootic disease in gilthead sea bream aquaculture, leading to significant morbidity and mortality due to its impact on host haemostasis, immune function, and lipid metabolism (Antonelli et al. 2010).

Although the antiparasitic potential of several essential oils (EOs) and their derived compounds against *S. chrysophrii* has been demonstrated *in vitro* (Sitjà-Bobadilla et al. 2006; Mladineo et al. 2021), their specific mechanisms of action on the parasite remain poorly understood. In particular, limited data are available regarding the direct lethal and sublethal effects of these compounds on key anatomical structures of the worm. To address this gap, a subset of parasites previously exposed to selected EOs (Pirollo et al. 2025) was processed for further morphological evaluation using scanning electron microscopy (SEM) and histological analysis.

In general, the tegument or neodermis and the associated structures of parasitic flatworms show considerable variation in their organization and fundamental functions. In polyophistocotylean monogeneans, the tegument represents a highly specialized interface that plays multiple roles in external body support, protection, attachment, nutrient exchange, and host-parasite interaction (Smyth 1984; Brennan and Ramasamy 1996; Santos and Lanfredi 2000). This region is richly supplied with nerve endings and glandular ducts and contains several superficial structures specialized for sensory perception and attachment to the host surface. Moreover, the tegument is anatomically connected to other organ systems, including the excretory outlets, the neural network, and the body wall musculature (Valigurová et al. 2021). The outer surface typically consists of a continuous syncytial layer covered by a thin glycocalyx, which likely contributes to protection against host immune and enzymatic activity (Brennan and Ramasamy 1996; Santos and Lanfredi 2000). Prominent folds, transverse ridges, and microvillous-like projections confer a corrugated appearance under light and electron microscopy and substantially increase the exposed area, thereby enhancing mechanical stability on the gill epithelium and facilitating nutrient absorption (Threadgold 1984; Cohen et al. 2001; Antonelli et al. 2010; Poddubnaya et al. 2015). Beneath the syncytial layer lies a well-developed musculature composed of circular, longitudinal, and diagonal fibers, which provide structural support and motility to the tegument.

Ultrastructural studies further reveal that the tegument is connected to subtegumental cell bodies via cytoplasmic extensions interwoven with muscle fibers, reflecting its highly dynamic and metabolically active nature (Santos and Lanfredi 2000; Cohen et al. 2001).

In *S. chrysophrii*, these general characteristics are particularly well developed. The lining of the oral cavity is folded and densely covered with microvilli and uniciliated structures, while the genital atrium exhibits concentric folds bordered by numerous microvilli.

The haptor, which is also covered by the continuous syncytial tegument, extends for more than half the total body length, and consists of two longitudinal rows of clamps supported on a cushion-like based on its ventral surface. The rows of clamps are similar in size and may comprise up to 72 pairs in mature subjects. This deeply folded and muscular tegumental region lacks sensory organs and structures and is highly specialized for mechanical attachment through these clamps (Antonelli et al. 2010; Mladineo et al. 2023).

The present study aims to characterize, through SEM and histological analyses, the ultrastructural alterations induced by these treatments, in order to elucidate potential mechanisms underlying their antiparasitic effects on *S. chrysophrii*.

## Materials and methods

Adult *S. chrysophrii* specimens used for morphological and ultrastructural analyses were obtained from the *in vitro* exposure trials previously described by Pirollo et al. (2025). Briefly, adult parasites were collected from experimentally infected gilthead sea bream (*Sparus aurata*) maintained at the Instituto de Acuicultura Torre de la Sal (IATS-CSIC, Spain), and exposed in 24-well plates (10 worms per well) to different concentrations of nine pure essential olis (EOs), namely cinnamon *Cinnamomum zeylanicum*, oregano *Origanum vulgare*, thyme *Thymus vulgaris*, rosemary *Rosmarinus officinalis*, peppermint *Mentha piperita*, lavender *Lavandula angustifolia*, tea tree *Melaleuca alternifolia*, lemon *Citrus limon* and blue gum *Eucalyptus globulus* and one commercial blend (Mix Gill), sourced from GreenVet, (Forlì, Italy), under controlled laboratory conditions (20 °C) for up to 24 h. All EOs were dissolved in dimethyl sulfoxide (DMSO) and tested at eight two-fold serial dilutions ranging from 7.8 to 500 ppm (mg/L), referring to the amount of whole EO preparation added to the exposure medium, as fully detailed in Pirollo et al. (2025). For each EOs a control group exposed to 0.1% DMSO has been included. Parasite viability was monitored at regular intervals (1 h, 2 h, 4 h, 6 h, 20 h and 24 h post-exposure) by observing spontaneous movements; worms not moving spontaneously were gently stimulated with a needle and classified as dead when no response was observed after 10 s while worms responding only with weak, slow, or spasmodic movements were classified as moribund.

During the *in vitro* experimental procedures, at each time point, up to five adult *S. chrysophrii* specimens found dead or moribund and their respective controls were removed from the wells and immediately preserved in 4% neutral-buffered formalin for histology, or in 2.5% glutaraldehyde in 0.1 M sodium cacodylate buffer (pH 7.2) overnight at 4 °C for SEM.

A total of 170 specimens (including controls) were preserved and processed for histology and SEM: approximately 82 were examined by histology and 76 by SEM. Specimens were collected across all ten EO treatments at concentrations ranging from 62.5 to 500 ppm and at multiple time points (1, 2, 6 and 20 h post-exposure), depending on treatment efficacy and the availability of dead or moribund worms. This selection approach means that the described lesions represent severe endpoint damage and should be interpreted accordingly. Formalin-fixed samples were processed for routine resin histology. Section  (4 μm) were mounted on Superfrost™ Plus slides (Menzel-Gläser, Braunschweig, Germany) and stained with Giemsa (Piazzon et al. 2018) for anatomical observations of the parasites.

Glutaraldehyde-fixed specimens were washed three times in the same buffer at 15-min intervals, post-fixed in 1% osmium tetroxide, dehydrated through a graded ethanol series, and dried in hexamethyldisilazane. The specimens were then mounted on aluminum stubs, sputter-coated with gold–palladium, and examined using a Phenom XL G2 Desktop SEM operating at 5 kV (protocol modified after Tedesco et al. 2021; 2022).

## Results

The tested EOs showed a marked variability in their antiparasitic efficacy, allowing a ranking based on their antiparasitic potency. This ranking is based on the quantitative LD_50_ values calculated across the full concentration range and multiple time points, as reported and statistically analyzed in Pirollo et al. (2025).

*C. zeylanicum*, *O. vulgare*, *T. vulgaris* and the blend named Mix gill were identified as the most effective, with short-term LD_50_ values below 100 ppm within 2 h of exposure (14, 54, 64 and 81 ppm respectively), followed by *R. officinalis*. Intermediate activity was observed for *M. piperita* (LD_50_ 134 ppm at 2 h, dropping to 6 ppm after 12 h), whereas *L. angustifolia*, *M. alternifolia*, *C. limon*, and *E. globulus* displayed comparatively lower lethality, with short-term LD_50_ values ranging from 265 to 353 ppm (Pirollo et al. 2025).

Overall, the control samples displayed the expected histological and ultrastructural features of normal adult *S. chrysophrii* specimens.

**Fig. 1:**
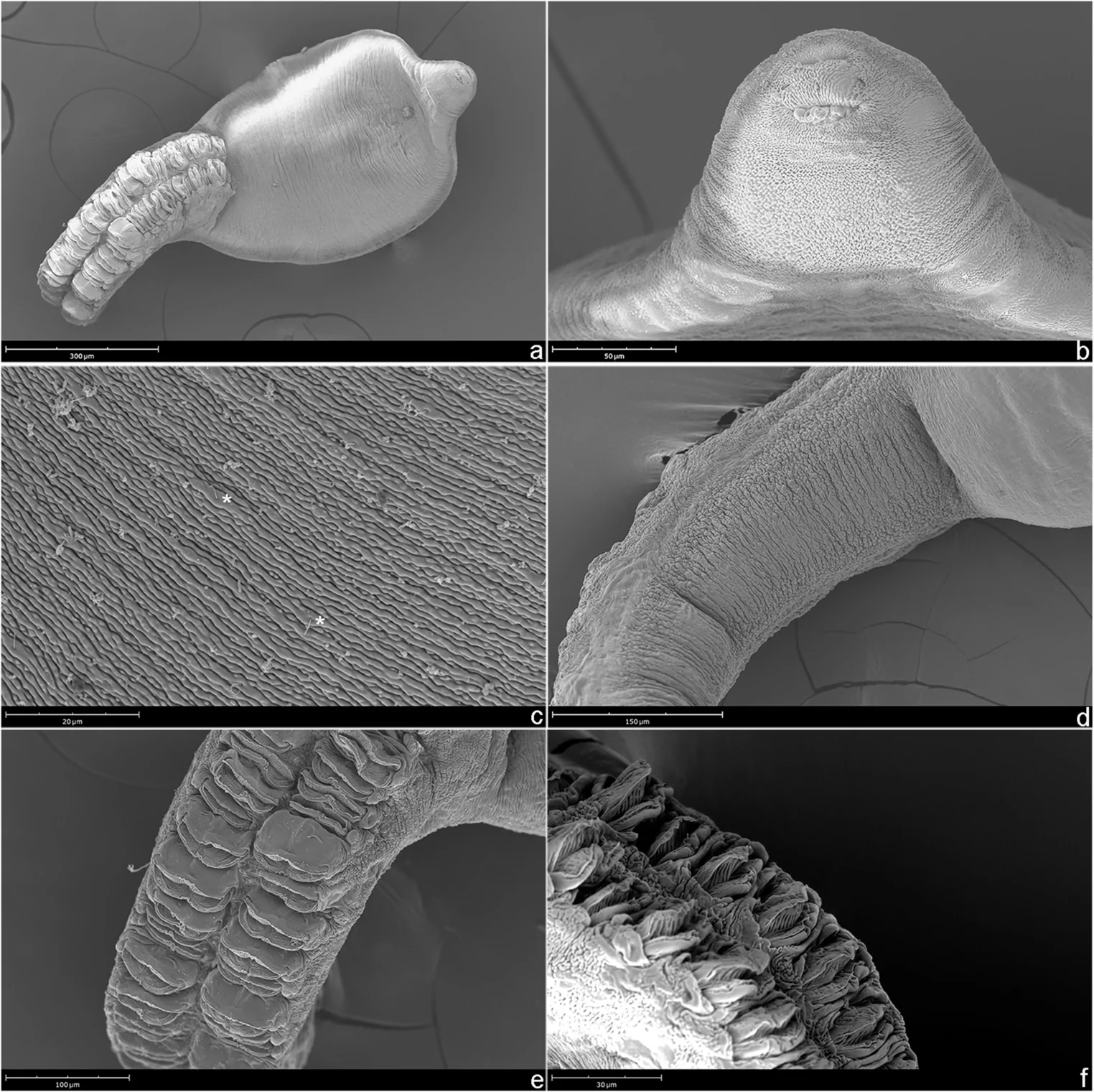
SEM micrographs of control specimens of *Sparicotyle chrysophrii*, showing (**a**) overall intact body structure (scale bar = 300 μm); (**b**) well-preserved structures of the anterior end (scale bar = 50 μm); (**c**) and (**d**) normal appearance of the tegument characterized by fine ridges (scale bar = 20 μm and 150 μm); (**e**) and (**f**) robust and symmetrical clamps with clearly defined segmentation and anchoring surfaces (scale bar = 100 μm and scale bar = 30 μm)

Analysis by SEM showed that control specimens exposed to 0.1% DMSO exhibited well-preserved morphology, with clamps and tegumental structures intact (Figs. 1a-f). The clamps appeared robust and symmetrical (Figs. 1e, f), with clearly defined segmentation and anchoring surfaces (Fig. 1f). The tegument showed a continuous surface characterized by fine transverse ridges and microstructural details, such as dome-shaped papillae bearing a single sensory cilium, particularly distributed in the cephalic region and around the oral cavity and potentially involved in environmental sensing and host localization (Brennan and Ramasamy 1996; Antonelli et al. 2010); these observed features are indicative of normal integrity and the absence of damage (Figs. 1b, c).

**Fig. 2:**
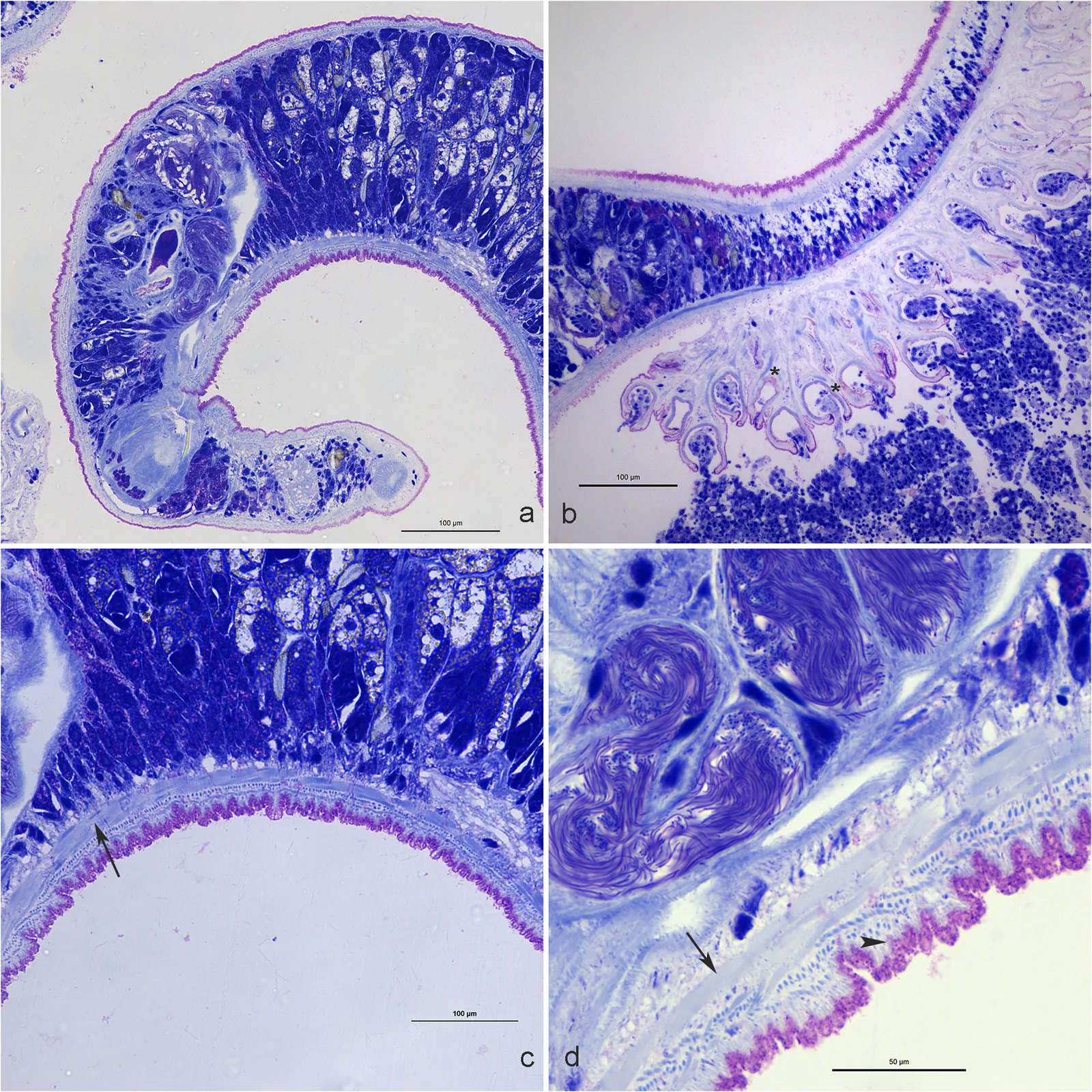
Histological section of the untreated *Sparicotyle chrysophrii* (Giemsa): (**a**) Anterior part of the parasite (scale bar = 100 μm); (**b**) Posterior part showing the haptor with clamps, intact peduncle, median sclerite (asterisks), and covered by the tegument (scale bar=100 μm); (**c**) High magnification of the tegument region in the anterior extremity, showing the thin, continuous neodermis consistent with its syncytial structure and the underlying musculature layers (arrow) (scale bar = 100 μm); (**d**) Higher magnification highlighting the neodermis (arrow head) and the underlying circular and longitudinal muscle layers (arrow) (scale bar = 50 μm)

Histological sections of untreated *S. chrysophrii* revealed well-preserved tissue architecture (Figs. 2a-d). The outer neodermis was clearly distinguishable as a thin, continuous layer lining the parasite’s surface, consistent with its syncytial organization (Fig. 2c). Beneath the neodermis, two distinct muscle layers were visible: an outer circular layer and an inner longitudinal layer, both densely stained and structurally organized (Fig. 2d). Clamp structures were also identifiable, appearing as specialized, well-defined attachment organs. Their peduncle, the connection to the tegument and the median sclerite support were intact, with no evidence of detachment or deformation (Fig. 2b).

After *in vitro* exposure to 500 ppm of EOs for 1 h, the four most effective EOs (*C. zeylanicum*, *O. vulgare*, *T. vulgaris* and *Mix Gill*) resulted in 100% parasite mortality across all replicate wells, as documented in Pirollo et al. (2025). The remaining EOs required longer exposure times or higher concentrations to achieve comparable lethality.

**Fig. 3:**
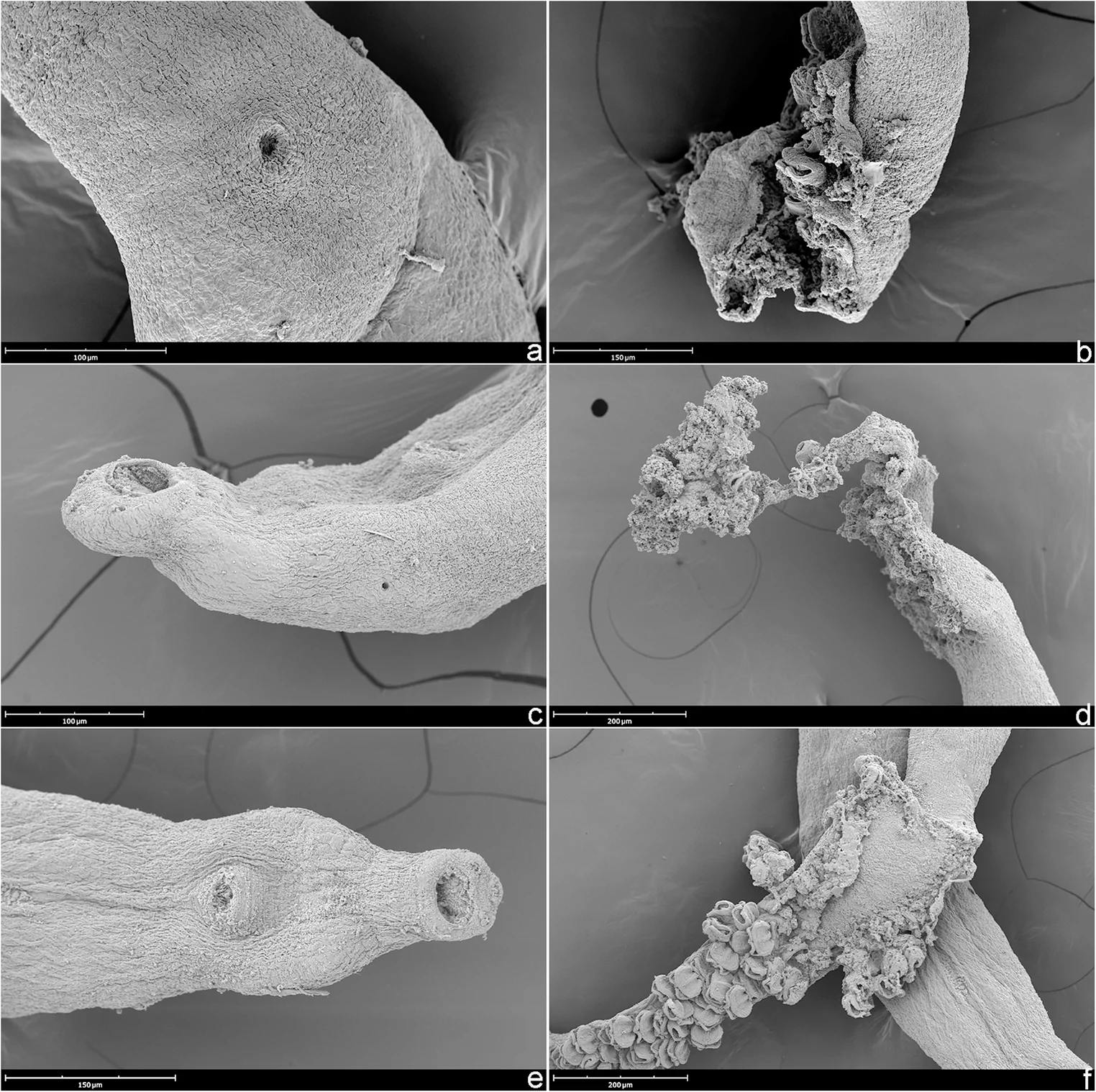
SEM micrographs of specimens of *Sparicotyle chrysophrii* following *in vitro* exposure to 500 ppm of EOs for 1 h (**a**) tegument alterations (scale bar = 100 μm) and (**b**) haptor disruption after exposure to *C. lemon* EO (scale bar = 150 μm); (**c**) tegument alterations (scale bar = 100 μm) and (**d**) complete loss of clamps in the haptor after exposure to *M. piperita* EO (scale bar = 200 μm); (**e**) tegument alterations (scale bar = 150 μm) and (**f**) partial loss of clamps and alteration of clamp organization in the haptor after exposure to *T. vulgaris* EO (scale bar = 200 μm)

Scanning electron microscopy revealed extensive structural damages across specimens. The images presented are representative examples of alterations consistently observed across multiple specimens exposed to the same treatment conditions, and some examples are reported in the Fig. 3.

Following exposure to 500 ppm for 1 h, the tegument exhibited clear signs of disruption, including surface roughening, and the presence of erosions and deep wrinkles (Figs. 3a, c, e). Haptor disruption with partial or complete detachment of the clamps was observed particularly with exposure to *C. lemon*, *M. piperita* and *T vulgaris* EOs (Figs. 3b, d, f). The superficial layers of the tegument were visibly compromised, showing diffuse erosion and detachment. The anterior region appeared slightly deformed, with collapsed or irregular contours (Figs. 3c, e).

**Fig. 4:**
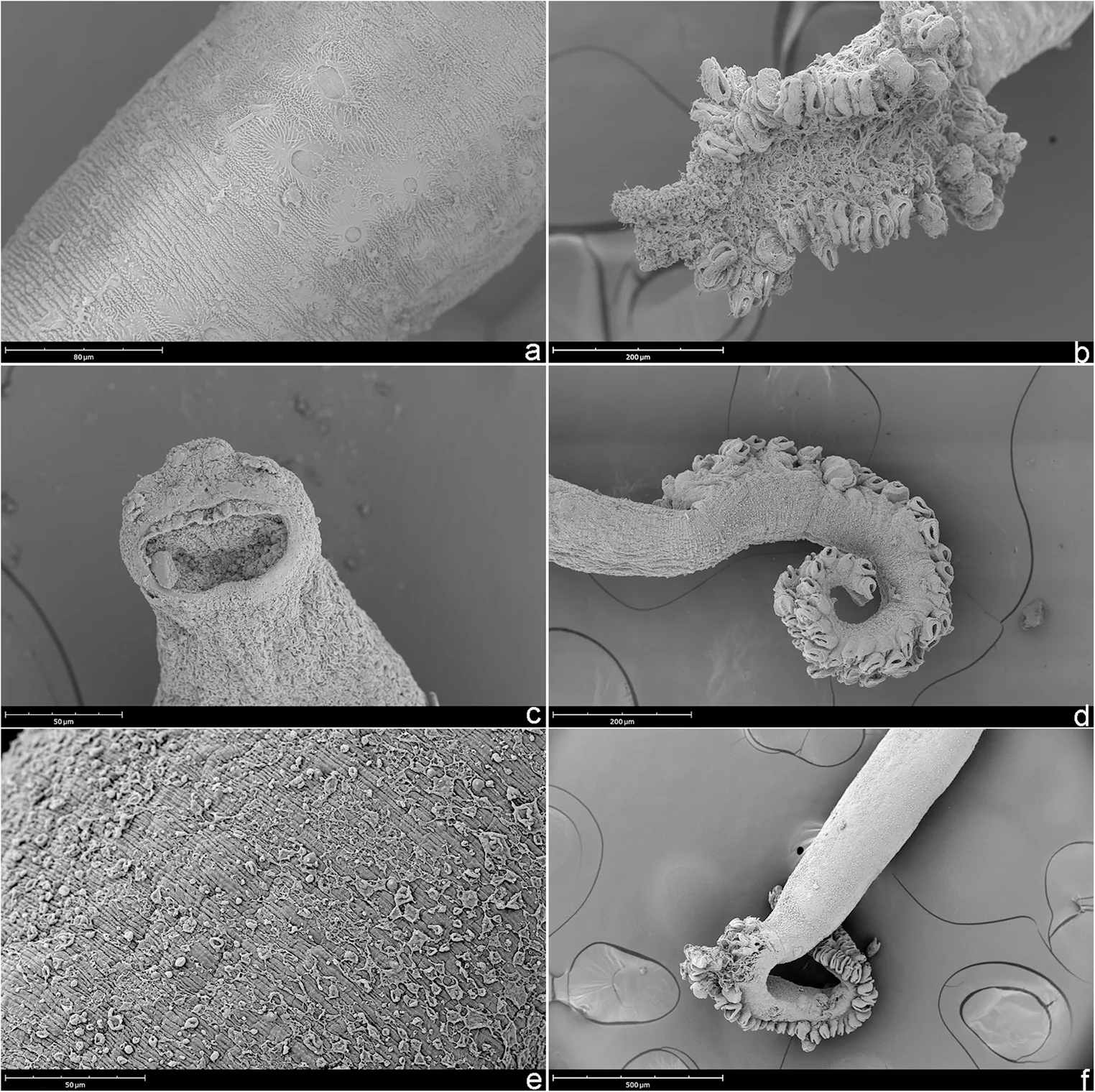
SEM micrographs of specimens of *Sparicotyle chrysophrii* following *in vitro *exposure to 250 ppm of EOs (**a**) tegument alteration with diffuse erosion (scale bar = 50 μm) and (**b**) haptor disruption with partial loss of clamps after exposure to *R. officinalis* EO for 2 h (scale bar = 200 μm); (**c**) deformation of anterior end with severe tegument damage (scale bar = 50 μm) and (**d**) partial alteration of clamp organization after exposure to *O. vulgare* EO for 1 h (scale bar = 200 μm); (**e**) tegument reactivity (scale bar = 50 μm) and (**f**) partial loss of clamps and alteration of clamp organization in the haptor after exposure to *C. zeylanicum* EO for 1 h (scale bar = 500 μm)

**Fig. 5:**
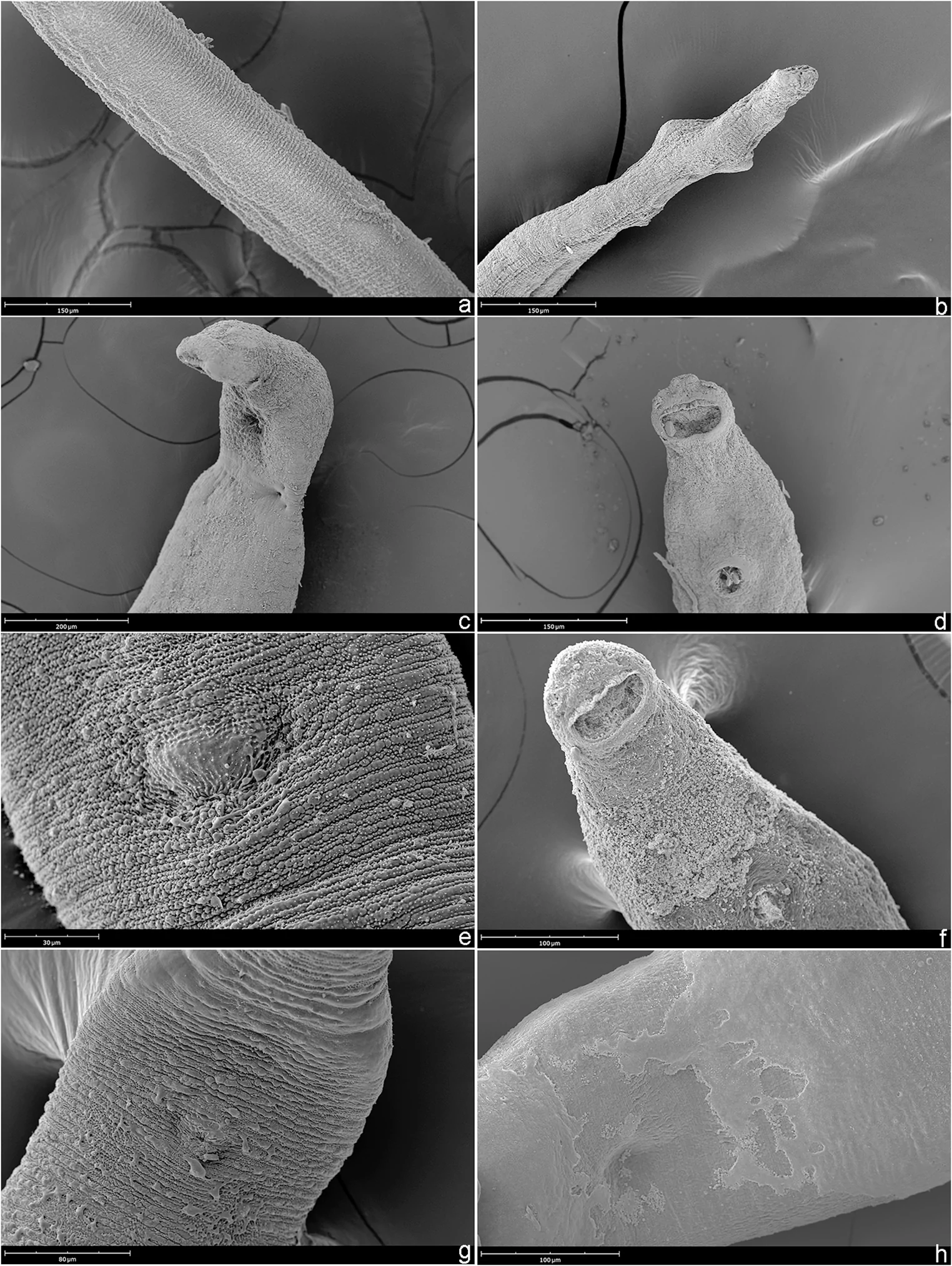
SEM micrographs of specimens of *Sparicotyle chrysophrii* following *in vitro* exposure to different concentrations of *C. zeylanicum* (**a**, **c**, **e**, **g**) and *O. vulgare* (**b**, **d**, **f**, **h**) for 1 h. (**a**) and (**b**) deformation and severe alteration of anterior region and tegument surface (500 ppm) [scale bar = 150 μm]; (**c**) [scale bar = 200 μm] and (**d**) less severe deformation of anterior end with tegument alteration (250 ppm) [scale bar = 150 μm]; (**e**) [scale bar = 30 μm] and (**f**) high reactivity of the tegument, particularly in the anterior region (125 ppm) [scale bar = 300 μm]; (**g**) reactive [scale bar = 50 μm] and (**h**) eroded areas of the outer tegument, with overall preserved structure (62.5 ppm) [scale bar = 100 μm]

Parasite specimens collected after 1–2 h of *in vitro* exposure to 250 ppm of EOs showed structural alterations primarily affecting the outer tegumental layers and clamp architecture, with lesions comparable in nature but generally less advanced than those observed at 500 ppm, consistent with a concentration-dependent effect (Fig. 4). Several areas of erosion and reactivity were observed across the tegument, particularly in the outer layers and subtegumental regions (Figs. 4a, e). Clamps showed partial detachment from the underlying structure and loss of their typical morphology, slightly deformed or curled (Figs. 4b, d, f). These observations are further supported by the Fig. 5, which clearly illustrates the progressive tegumental damage observed in parasites exposed to *C. zeylanicum* (Figs. 5a, c, e, g) and *O. vulgare* (Figs. 5b, d, f, h) at increasing concentrations, from 62.5 to 500 ppm, collected after 1 h of *in vitro* exposure.

**Fig. 6:**
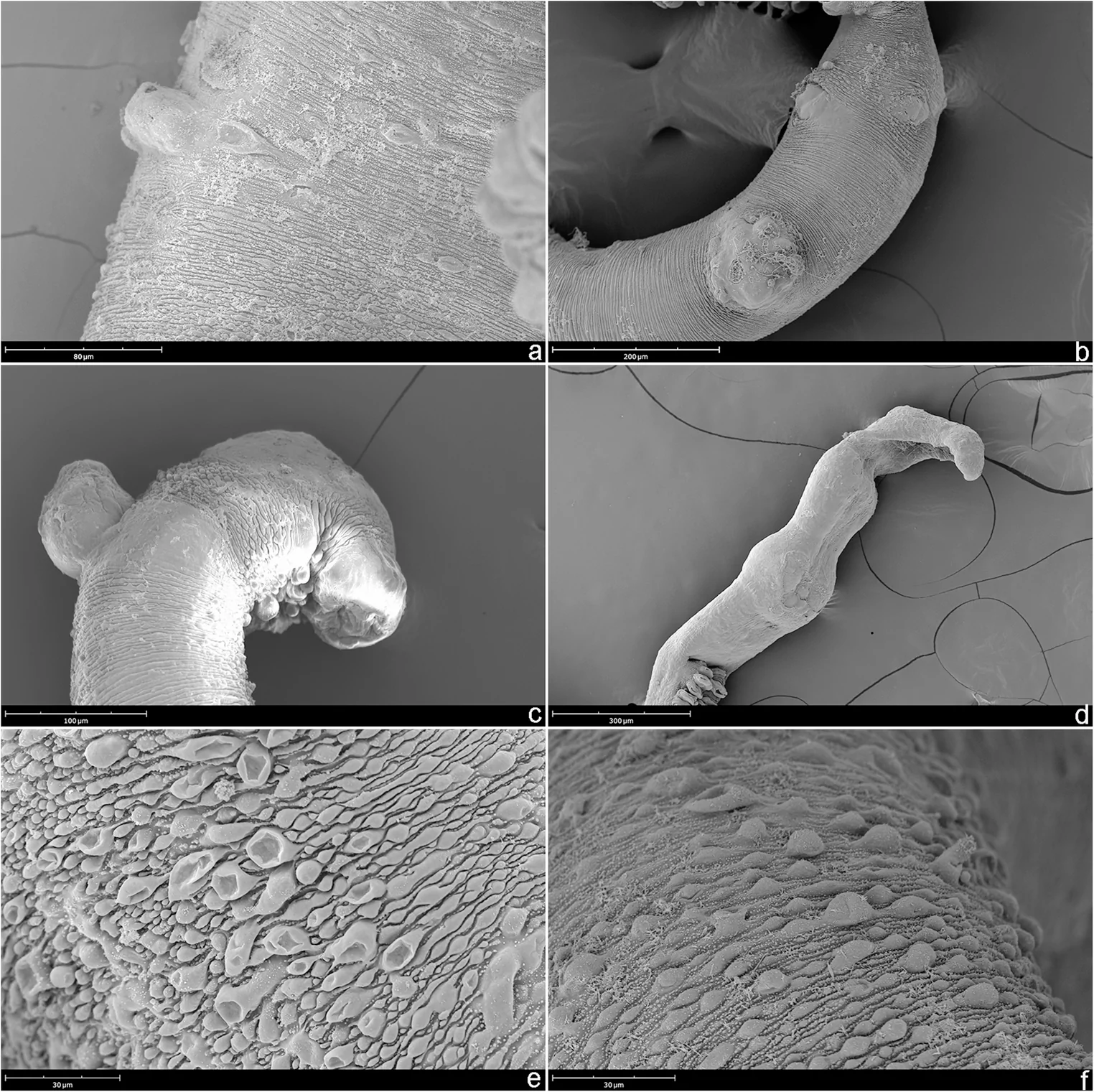
SEM micrographs of specimens of *Sparicotyle chrysophrii* following *in vitro* exposure to different concentrations of EOs for up to 20 h. (**a**) localized swelling of the tegument after exposure to *L. angustifolia* (250 ppm) for 20 h (scale bar = 80 μm); (**b**) swelling and deformation after exposure to *R. officinalis* (125 ppm) for 6 h (scale bar = 200 μm); (**c**) swelling and deformation after exposure to Mix gill (62.5 ppm) for 6 h (scale bar = 100 μm); (**d**) deformation and tegument alteration after exposure to *O. vulgare* (62.5 ppm) for 6 h (scale bar = 300 μm); (**e**) intense tegument reaction with diffused vesicle-like formations (“budding”) after exposure to *T. vulgaris* (62.5 ppm) for 6 h (scale bar=30 μm); (**f**) initial stages of tegument reaction after exposure to *L. angustifolia* (250 ppm) for 20 h (scale bar = 30 μm)

It was also observed that not only concentration, but also exposure time significantly influenced the extent of tegumental damage. As shown in Fig. 6, parasites collected after 6–20 h of exposure exhibited clear morphological alterations, consistent with a progressive parasite degeneration.

In particular, localized swelling of the tegument was frequently observed, especially with *L. angustifolia*,* R. officinalis*, Mix gill and *O. vulgare* EOs (Figs. 6a-d), often accompanied by extensive deformation, indicating a structural damage of the parasite (Figs. 6b-d). The image also revealed the presence of diffused vesicle-like formations (“budding”), indicative of intense tegumental reaction (Figs. 6e, f) or degenerative response preceding the extensive erosion seen in specimens exposed to higher concentrations.

**Fig. 7:**
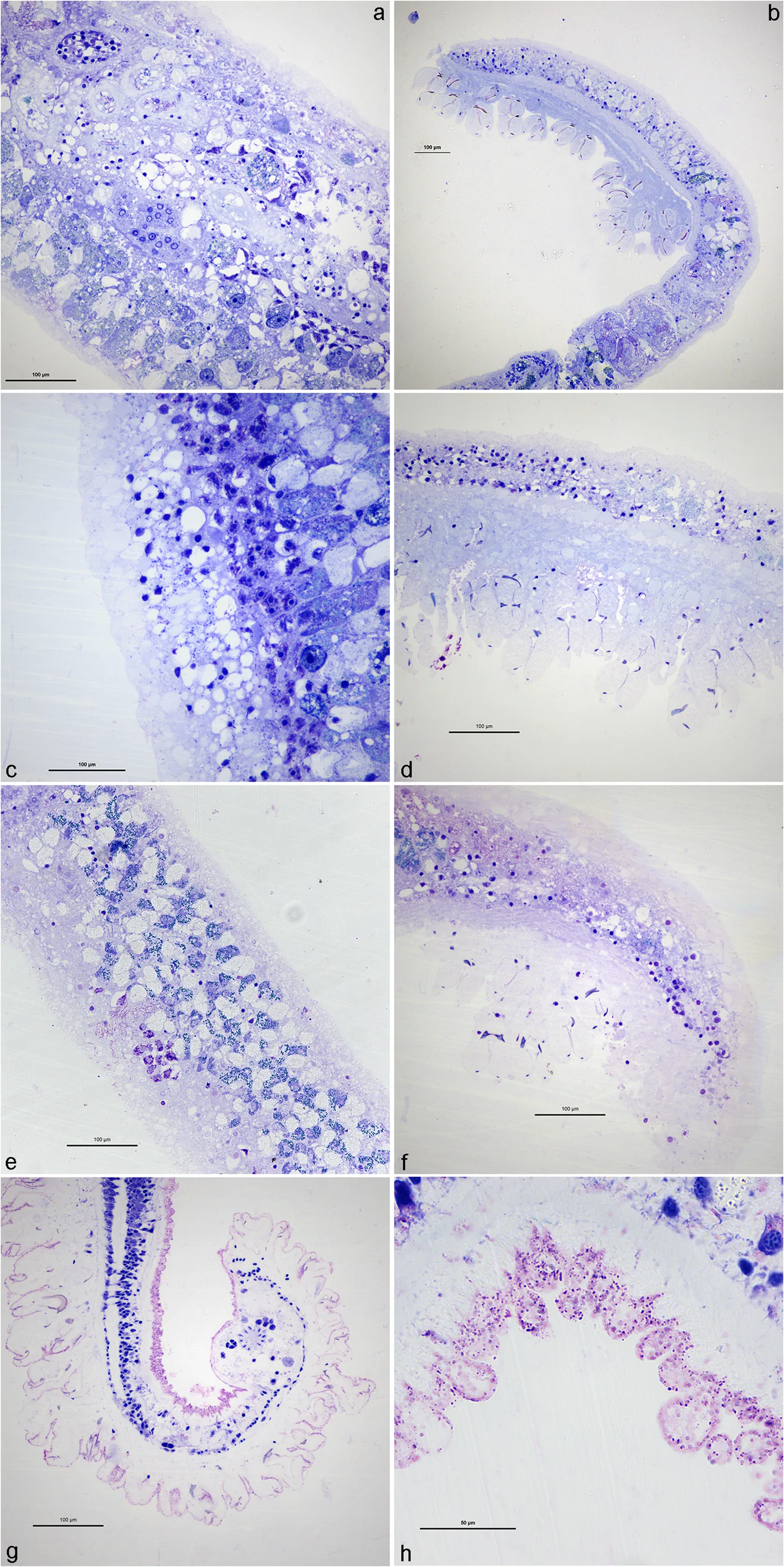
Histological section of *Sparicotyle chrysophrii* exposed to different concentration of *Thymus vulgaris* EO (Giemsa): (**a**, **b**) specimens treated with 500 ppm showing severe tegumental disruption, extensive vacuolization, necrosis and disorganization of the parenchyma; damage to the clamps (scale bar = 100 μm); (**c**, **d**) specimens treated with 250 ppm, showing marked tissue disorganization and swelling of clamp structures (scale bar = 100 μm); (**e**, **f**) specimens treated with 125 ppm, showing lesions comparable to those produced with higher product concentrations (scale bar = 100 μm); (**g**, **h**) specimens treated with 62,5 ppm, showing better defined tegumental architecture, with localized swelling and early budding-like surface protrusions (scale bar = 100 μm and 50 μm)

Histological sections of *S. chrysophrii* exposed to 500 ppm of EOs for 1 h showed severe degenerative alterations affecting both the tegumental and muscular structures. The tegument appeared markedly disrupted, with loss of the normal layered organization and indistinct boundaries between the neodermis and underlying tissues. Extensive vacuolization and cytoplasmic rarefaction were evident throughout the parenchyma, indicative of edema and advanced cellular degeneration. The general tissue architecture was highly disorganized. Clamp structures exhibited pronounced histopathological changes, including rarefaction of the peduncle, loss of internal organization, partial detachment from the surrounding parenchyma with remaining as the only clearly recognizable structure. (Figs. 7a, b). In several specimens, clamps displayed irregular contours and reduced cellular density. Overall, these lesions indicate that exposure to EOs at this concentration induces acute structural collapse, compromising both the parasite’s tegumental integrity and its attachment apparatus, ultimately leading to mortality.

At 250 ppm, the histological appearance of the parasite was largely comparable to that observed at the higher concentration. The specimens exhibited similar tegumental alterations, including extensive vacuolization, loss of tissue organization, and disruption of the outer layers. Clamp structures also showed the same pattern of structural damage, with swelling and partial detachment from the body wall (Figs. 7c, d). Overall, no substantial qualitative differences were observed between the two concentrations, suggesting that even at 250 ppm, the EO induced severe tissue degeneration consistent with the lethal effects documented at higher doses.

At 125 ppm, histological alterations were still comparable to those observed at higher concentrations, although inconsistent staining made detailed evaluation still difficult (Figs. 7e, f). At 62.5 ppm, staining quality improved and the general tissue organization was better defined, with the tegumental architecture beginning to recover its stratified appearance. Localized swelling was observed in the outer layers, together with early signs of surface protrusions resembling *budding* or vesicle-like formations, consistent with the ultrastructural alterations documented by SEM at the same concentration (Figs. 7g, h). These lesions appeared as initial degenerative changes preceding the extensive erosion and tissue collapse seen at higher doses.

## Discussion

The present morphological investigation provides new insights into the structural damage induced by different EOs, with emphasis on tegumental and clamp alterations observed through SEM and histological analyses.

In addition to documenting treatment-related alterations, this study also emphasizes the limited availability of detailed morphological descriptions of *S. chrysophrii*, particularly concerning the tegument and clamp structures observed under SEM and histology. This scarcity of baseline information complicates the interpretation of experimental lesions and highlights the need for further morphological characterization of this species.

Overall, both SEM and histological examinations demonstrated that exposure to EOs causes severe tegumental disruption, detachment of the syncytial layer, surface erosion, and generalized tissue degeneration. The extent of damage appeared to correlate not only with EO concentration but also with exposure time, as previously reported for other monogenean species. Particularly, EOs at 500 ppm exerted a rapid and potent parasiticidal effect, compromising the parasite’s external architecture and viability. Furthermore, our findings suggest that prolonged exposure to lower concentrations of EOs, although not inducing rapid mortality as observed at higher doses, triggers a gradual yet irreversible sequence of structural alterations. Over time, this cumulative damage compromises the tegument’s protective function, eventually leading to death. This evidence highlights a time-dependent toxic effect, where sustained sublethal exposure can be equally detrimental through progressive tegumental deterioration.

In monogeneans, the tegument plays a key role in protection, osmoregulation, attachment, and host–parasite interactions (Brennan and Ramasamy 1996; Santos and Lanfredi 2000; Valigurová et al. 2021). Disruption of this multifunctional barrier likely compromises parasite homeostasis and increases susceptibility to host immune responses.

Our findings are in agreement with previous ultrastructural studies describing EO-induced lesions in other monogeneans parasites. For instance, Ling et al. (2015) reported that cinnamaldehyde caused significant tegumental disruption in *Dactylogyrus intermedius* Wegener, 1910, with wrinkling, holes, and nodular structures. Similarly, Zhang et al. (2020) observed in *Gyrodactylus kobayashii* Hukuda, 1940 exposed to curdione (*Curcuma zedoaria*) a progressive, concentration-dependent disappearance of microvilli and the appearance of multiple small perforations, features remarkably similar to those documented in the present study. Comparable surface alterations, such as wrinkling, detachment, and tegumental collapse, have also been described in *Anacanthorus spathulatus* Kritsky, Thatcher & Kayton, 1979, *Mymarothecium boegeri* Cohen & Kohn, 2005, and *Notozothecium janauachense* Belmont-Jégu, Domingues & Martins, 2004, parasites of the species *Colossoma macropomum*, following *in vitro* exposure to several plant-derived compounds, including lemongrass (*Cymbopogon citratus*) (Gonzales et al. 2020), *Copaifera reticulata* oleoresin (Malheiros et al. 2020), pepper (*Piper* spp.) extracts (Alves et al. 2021), *Lippia alba* (Tavares-Dias et al. 2021), shell ginger (*Alpinia zerumbet*) (Luz et al. 2021).

While the precise molecular targets of EOs in fish parasites remain unclear, their lipophilic nature suggests a direct interaction with cell membranes, leading to increased permeability and loss of structural integrity (Bakkali et al. 2008; Tavares-Dias 2018). The ability of EO constituents to penetrate the lipid-rich tegument may also disturb subtegumental structures, causing leakage of cellular contents, cytoplasmic vacuolization, and general tissue rarefaction. Although direct confirmation in *S. chrysophrii* is still lacking, these pathways may contribute to the structural degeneration documented in our study, particularly the vacuolization and tissue disorganization revealed histologically and the severe erosion observed by SEM. Such lesions likely reflect a progressive loss of osmotic balance and structural cohesion rather than specific intracellular targeting. Additionally, oxidative stress and mitochondrial dysfunction have been proposed as contributing factors in other parasitic taxa, possibly enhancing the overall cytotoxic effect (Sharifi-Rad et al. 2017). The multi-component nature of EOs also implies potential synergistic effects among different molecules, acting on multiple cellular targets simultaneously and enhancing antiparasitic efficacy. However, further ultrastructural and biochemical investigations are required to elucidate these mechanisms in *S. chrysophrii*.

From a methodological perspective, this preliminary work confirms the relevance of SEM as a sensitive and informative approach for assessing morphological damage in parasites treated with EOs. Conversely, histological evaluation proved more challenging due to sample fragility, potential interference of EO residues with fixation and staining, and difficulty in distinguishing lesion severity between concentrations and exposure times.

During histological processing, we encountered technical challenges related to staining consistency. In particular, slides prepared from parasites exposed to higher concentrations of EOs showed progressive fading of stain intensity over time. This phenomenon appeared to correlate with the treatment itself, suggesting a possible interference between the EO residues and the fixation or staining process. As a result, the overall quality of the histological slides was variable, especially at higher concentrations, limiting the number of interpretable samples.

These limitations suggest that histology, while valuable, should be complemented by other ultrastructural approaches to obtain a more reliable interpretation of EO-induced pathology. Furthermore, it should be acknowledged that specimens selected for SEM and histological examination were exclusively dead or moribund worms, which introduces a selection bias: the described alterations represent severe endpoint damage and may not capture the full spectrum of sublethal effects.

To gain deeper insight into the mechanisms of EO antiparasitic activity, future studies should integrate morphological, biochemical, and molecular approaches. In particular, transmission electron microscopy (TEM) could help elucidate subcellular alterations and identify potential targets within the tegumental or muscular systems. Moreover, isolating and testing individual EO components, or assessing their synergistic combinations, may clarify the relative contribution of each molecule to the overall antiparasitic effect.

In conclusion, this study provides detailed morphological evidence of the destructive effects of EOs on *S. chrysophrii*, contributing new ultrastructural data to the limited existing literature on this parasite.

Our SEM and histological findings collectively support a time and concentration-dependent mechanism of tegumental and structural collapse, which may underlie the lethal activity observed in vitro. These results encourage further exploration of plant-derived compounds as potential eco-friendly alternatives for monogenean control in aquaculture, while highlighting the critical role of ultrastructural analyses in understanding their mode of action.

## Data Availability

All the data that support the findings of this study are available from the corresponding author.
